# Enoxaparin Versus Dalteparin for Venous Thromboembolism Prophylaxis in Hip and Knee Arthroplasty: A Systematic Review

**DOI:** 10.7759/cureus.93928

**Published:** 2025-10-06

**Authors:** Osman Haji, Sarah Davidson, Chimela Nwamba, Shyamala Manibalan

**Affiliations:** 1 General Practice, King's College Hospital NHS Foundation Trust, London, GBR; 2 Emergency Department, Croydon Health Services NHS trust, London, GBR; 3 General medicine, Croydon Health Services NHS Trust, London, GBR; 4 Geriatric Medicine, King's College Hospital NHS Foundation Trust, London, GBR

**Keywords:** dalteparin, enoxaparin sodium, low-molecular-weight heparin (lmwh), thromboprophylaxis, total hip arthroplasty (tha), total knee arthoplasty (tka), venous thromboembolism (vte)

## Abstract

Venous thromboembolism (VTE) is a serious postoperative complication in patients undergoing total hip or knee arthroplasty, with both pulmonary embolisms and deep vein thromboses being major causes of preventable morbidity and mortality. Low-molecular-weight heparins such as enoxaparin and dalteparin are widely used for thromboprophylaxis, but direct comparative data are limited. We aimed to do a systematic review of the literature to compare the efficacy and safety of enoxaparin and dalteparin in preventing VTE following total hip or knee replacement surgery. A systematic literature search was conducted in MEDLINE, PubMed, Embase, and the Cochrane Library. After deduplication, 145 studies were screened, 28 full texts were assessed, and three papers were included. Eligible studies were randomised controlled trials (RCTs) or observational studies comparing enoxaparin and dalteparin in adult patients undergoing hip or knee arthroplasty, reporting VTE rates as the primary outcome. Risk of bias (ROB) was assessed using the Cochrane RoB 2 tool for randomised trials and the Newcastle-Ottawa Scale for observational studies. Three studies - one RCT and two retrospective cohort studies with a total of 1,167 patients - met the inclusion criteria. Across all studies, VTE incidence was low with no significant difference between enoxaparin and dalteparin. Some evidence suggested dalteparin may be associated with fewer bleeding events and lower transfusion rates, with one study also reporting cost savings. ROB was moderate in the observational studies, with some concerns in the RCTs. Enoxaparin and dalteparin appear similarly effective for VTE prevention after hip or knee arthroplasty. However, dalteparin may be associated with a lower bleeding risk and reduced transfusion rates, with potential cost savings. Current evidence is limited by the small number and quality of studies. Further high-quality randomised trials are needed to confirm these findings and inform clinical decision-making.

## Introduction and background

Total hip arthroplasty (THA) and total knee arthroplasty (TKA) are among the most commonly performed orthopaedic procedures worldwide [1]. As populations age and surgical techniques advance, the number of these procedures continues to rise [2], improving the quality of life for patients with severe osteoarthritis and other degenerative joint diseases [3]. However, despite their benefits, THA and TKA are associated with a substantial risk of postoperative venous thromboembolism (VTE), including deep vein thrombosis (DVT) and pulmonary embolism (PE) [4]. Without effective thromboprophylaxis, the incidence of VTE in this patient population may exceed 40-80%, with a proportion developing potentially fatal PE [5].

To mitigate this risk, clinical guidelines from major bodies such as the American College of Chest Physicians (ACCP) and the National Institute for Health and Care Excellence (NICE) recommend routine thromboprophylaxis in all patients undergoing major lower limb orthopaedic surgery [6]. Low-molecular-weight heparins (LMWHs) have long been a cornerstone of pharmacological prophylaxis due to their predictable pharmacokinetics, ease of subcutaneous administration, and favourable safety profile compared to unfractionated heparin (UFH) and vitamin K antagonists [7]. Enoxaparin and dalteparin are two commonly used LMWHs. Although both act primarily by inhibiting factor Xa, subtle pharmacokinetic and pharmacodynamic differences, such as anti-Xa to anti-IIa ratios, half-life, and tissue penetration, may influence clinical outcomes, including bleeding risk [8-11].

Despite their widespread use, direct head-to-head comparisons of enoxaparin and dalteparin in THA and TKA patients are limited and often heterogeneous. Much of the available evidence comes from small studies or retrospective analyses, leaving uncertainty regarding which LMWH is preferable in terms of VTE prevention, bleeding risk, and cost-effectiveness. Cost considerations are particularly relevant as formulary choices are often influenced by drug acquisition and administration costs, and even small per-patient differences may have substantial financial implications when scaled across large surgical populations [12].

Bleeding events, both major and minor, can lead to an array of issues. This can include delay in mobilisation, increased need for transfusions, and prolonged hospital stay [13,14]. Their definitions, therefore, require careful consideration to ensure comparability between studies. Direct comparative evidence between enoxaparin and dalteparin in this surgical population is somewhat limited, highlighting the need for a focused synthesis of available data. This review aims to summarise randomised controlled trials (RCTs) and observational studies directly comparing these agents in THA and TKA patients to clarify their relative efficacy, safety, and cost implications.

## Review

Methods

Search Strategy

A systematic search of MEDLINE, PubMed, Embase, and the Cochrane Library was conducted in April 2025 to identify studies comparing enoxaparin and dalteparin for VTE prophylaxis in patients undergoing hip or knee replacement. Search terms combined keywords and controlled vocabulary related to “enoxaparin”, “dalteparin”, “low-molecular-weight heparin”, “venous thromboembolism”, “deep vein thrombosis”, “pulmonary embolism”, “hip replacement”, and “knee replacement”. The Appendix provides examples of search strings used. Reference lists of relevant articles were also screened to identify additional studies.

Eligibility Criteria

Studies were eligible if they directly compared enoxaparin with dalteparin for VTE prophylaxis, included adult patients undergoing hip or knee replacement, reported clinical outcomes related to VTE (deep vein thrombosis, pulmonary embolism, or symptomatic VTE) and/or safety outcomes such as bleeding or transfusion, were published in English, and were RCTs or observational cohort studies. Exclusion criteria were non-English studies, case reports, case series, editorials, narrative reviews, and conference abstracts without full text, studies of VTE prophylaxis in populations other than hip or knee replacement (e.g., trauma, general surgery, or medical inpatients), and studies not reporting comparative outcomes between enoxaparin and dalteparin.

The studies remaining after deduplication were screened by title and abstract by three reviewers, with disagreements resolved by discussion and building consensus. All those not excluded at this stage were sought for full-text retrieval and were further assessed for eligibility. A Preferred Reporting Items for Systematic Reviews and Meta-Analyses (PRISMA) flow diagram was constructed to document the screening and selection process.

Data were extracted independently using a pre-designed form. Extracted information included study characteristics (author, year, country, setting, design), patient population (sample size, type of surgery), intervention details (dose, timing, duration of enoxaparin and dalteparin), and clinical outcomes (symptomatic VTE, deep vein thrombosis, pulmonary embolism, major bleeding, transfusion, and mortality).

RCTs were evaluated using the Cochrane Risk of Bias 2 (RoB 2) tool, and observational studies using the Newcastle-Ottawa scale. Discrepancies between reviewers were to be resolved by discussion and consensus.

Due to the limited number of studies, small sample sizes, and heterogeneity in design and reporting, a narrative synthesis of the results was performed. Where possible, event rates for enoxaparin and dalteparin were compared directly, but formal meta-analysis was not undertaken.

Results

Study Selection

**Figure 1:**
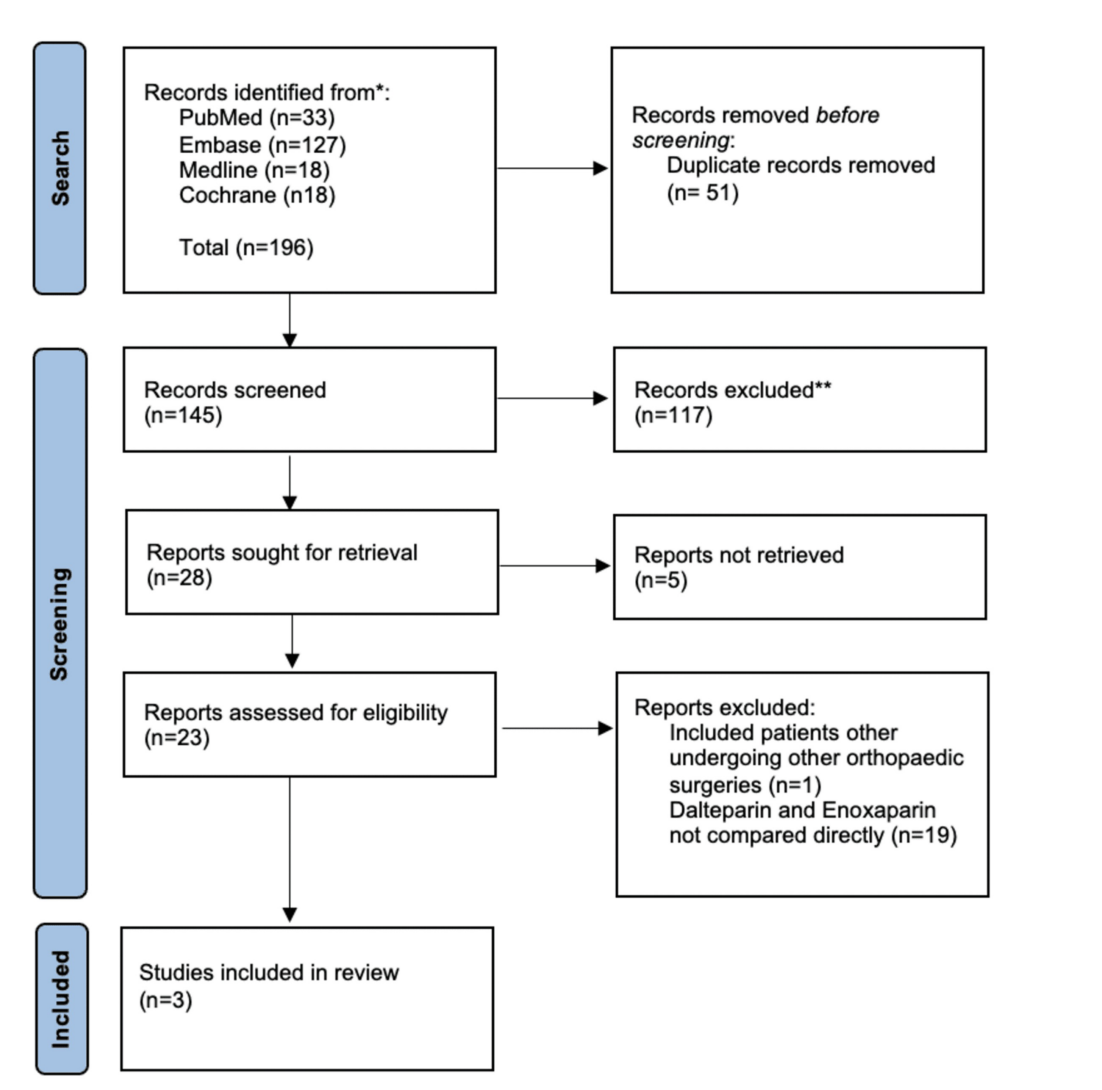
Preferred Reporting Items for Systematic Reviews and Meta-Analyses (PRISMA) flow chart

The initial search retrieved 145 papers. After the removal of duplicates, all titles and abstracts were screened. Twenty-eight articles were sought for full-text retrieval, of which three studies fulfilled the eligibility criteria and were included in the analysis. Figure 1 is a PRISMA flow diagram that summarises the study selection process.

Study Characteristics

The three included studies comprised one RCT and two retrospective observational cohorts, enrolling a total of 1,167 patients undergoing hip or knee replacement. The randomised trial of Biławicz et al. (2020) [15] enrolled 66 patients, while the observational studies of Krotenberg et al. [16] and Holden and Maceira [17] included 461 and 640 patients, respectively. Across studies, both hip and knee arthroplasties were represented, with variable proportions of each. Enoxaparin was administered as 40 mg subcutaneously once daily in the RCT, while dalteparin was given as 5,000 IU once daily. In the observational studies, the precise prophylactic regimens were less consistently reported, although both agents were used in routine clinical practice. The duration of prophylaxis also varied. 

Table 1 summarises the basic characteristics, methods, and results of the studies. Table 2 shows the Newcastle-Ottawa ratings for the two observational studies, while Table 3 shows the Cochrane RoB tool for the randomised study.

**Table 1:** Study characteristics and results

Study ID	Country/setting	Design	Population	Surgery mix (enoxaparin/daleparin)	Sample (total/enoxaparin/dalteparin)	Enoxaparin regimen	Dalteparin regimen	Prophylaxis duration	Follow-up duration	Primary outcomes reported	Symptomatic VTE (enoxaparin vs. dalteparin)	DVT/PE	Major bleeding	Transfusion (any)	Other notable findings
Biławicz et al., 2020 [15]	Poland; single-centre (Warsaw)	Randomised, prospective pilot (in-hospital prophylaxis)	Adults undergoing elective THR or TKR	17 THR + 17 TKR / 16 THR + 16 TKR	66 / 34 / 32	40 mg SC once daily; first dose 12 h pre-op; next ≥12 h post-op; in-hospital only	5,000 IU SC once daily; first dose 12 h pre-op; next ≥12 h post-op; in-hospital only	Not explicitly stated (in-hospital only)	3 months	Clinical/lab coagulation metrics; transfusion; VTE events	0/34 (0%) vs. 0/32 (0%)	0 / 0 in both arms	0 in both arms	17/34 (50%) enoxaparin vs. 8/32 (25%) dalteparin	Duke’s bleeding time was shorter with dalpearin on day 5 (p = 0.03).
Krotenberg et al., 2001 [16]	USA; Kessler Institute inpatient rehabilitation (post-THA/TKA)	Retrospective cohort (formulary switch)	Patients in inpatient rehabilitation after THA or TKA	Hip 48.5%/55%; knee 51.5%/45% (by arm)	461 / 161 / 300	Dose not fully specified; pricing assumptions used 30 mg dose units	Dose not fully specified; pricing assumptions used 2,500–5,000 IU dose units	During rehabilitation stay only	Rehabilitation stay only (post-discharge events not captured)	Symptomatic DVT (duplex-confirmed in symptomatic pts), bleeding (minor), costs	3/161 (1.9%) vs. 1/300 (0.3%); all distal DVT	Distal DVT only; PE not reported	0 (all bleeding events minor)	Not reported	Adjusted per-capita prophylaxis cost $129 lower with dalteparin
Holden and Maceira, 2015 [17]	USA; Albany Medical Center (THA/TKA)	Retrospective review (aspirin vs. pooled anticoagulants; agent-level counts)	Adults undergoing THA or TKA	71 hip + 82 knee (enoxaparin); 211 hip + 276 knee (DALT)	640 / 153 / 487	Not reported	Not reported	Not reported (per institutional order sets)	Events during index stay + readmissions within 35 days	Symptomatic VTE ("failure")	1/153 (0.65%) vs. 8/487 (1.64%)	Not reported	Not reported	Not reported	Non-pharmacologic prophylaxis used in >95% of patients; no fatal outcomes

**Table 2:** Newcastle-Ottawa assessment for the observational studies

Study	Representativeness of exposed cohort (★)	Selection of non-exposed cohort (★)	Ascertainment of exposure (★)	Outcome not present at start (★)	Comparability - important factor (★)	Comparability - additional factors (★)	Assessment of outcome (★)	Follow-up long enough (★)	Adequacy of follow-up (★)	Total (max 9)
Holden and Maceira, 2015 [17]	1	1	1	0	0	0	1	1	0	5
Krotenberg et al., 2001 [16]	1	1	1	0	1	0	1	0	1	6

**Table 3:** Cochrane risk of bias tool for the randomised study

Study	Outcome	D1. Randomization process	D2. Deviations from intended interventions	D3. Missing outcome data	D4. Measurement of the outcome	D5. Selection of the reported result	Overall risk of bias
Białawicz et al., 2020 [15]	Symptomatic VTE within three months	Some concerns	Some concerns	Some concerns	Some concerns	Some concerns	Some concerns

Narrative Syntheses

Paper 1: Krotenberg et al. (2001) [16] was a single-centre retrospective cohort built around a formulary switch (from enoxaparin to dalteparin) at an inpatient rehabilitation facility caring for patients after primary THA/TKA. The objective was to compare clinical effectiveness, safety, and dispensing costs of dalteparin versus enoxaparin during the rehabilitation stay.

Included patients had undergone THA or TKA at a Kessler institute hospital and, during the rehabilitation stay, received either enoxaparin or dalteparin (patients given ASA could still be included if they received enoxaparin or dalteparin in rehabilitation). Data were abstracted from medical records using a standard form (5% verification sample by physician authors).

Anticoagulant choice was non-random and determined by a formulary era. Precise doses during rehab were not uniformly documented in the paper, and as for the economic analysis, unit costs were anchored to 30 mg enoxaparin dose units and 2,500-5,000 IU dalteparin dose units (Red Book, 2000), in addition to a $5 per-dose pharmacy dispensing fee. Symptom-triggered duplex-confirmed DVT events during the rehab stay only (no routine screening) was measured as a proxy for efficacy. Clinically adjudicated bleeding events (with indicative haematology) were used to account for bleeding risk, although this was again limited to the rehabilitation stay, and post-discharge events were not captured, meaning the study may underestimate total event rates.

The total sample size included was 461 (enoxaparin 161; dalteparin 300). Comparative analyses used age-adjusted odds ratios, and the paper compared baseline characteristics to check for treatment-era differences.

The results (rehab-stay outcomes) are as follows: Three (1.9%) patients developed DVT with enoxaparin compared to just one (0.3%) with dalteparin. The age-adjusted odds ratio (dalteparin vs. enoxaparin) was 0.160 (95% CI 0.016-1.569). Bleeding was observed in six (3.7%) patients within the enoxaparin group compared to eight (2.3%) with dalteparin. The odds ratio for this was 0.634 (95% CI 0.209-1.922). The composite risk (the risk of developing DVT or bleeding, calculated from adding the percentages of bleeding and DVT events) was 5.6% in the enoxaparin arm and 2.7% in the dalteparin arm (odds ratio 0.471, 95% CI 0.178-1.247). Adjusted per-capita prophylaxis was $129 lower with dalteparin overall. Savings were varied by surgery type ($108 THA vs. $153 TKA). Authors emphasise cost differences alongside equivocal clinical differences due to low event counts.

Within a rehabilitation-only window (i.e., after the arthroplasty admission), event rates were low and confidence intervals wide. Point estimates favoured dalteparin for symptomatic DVT and bleeding, but precision was poor, and no clear superiority can be claimed. Importantly, the study does not report VTE events that occurred post-discharge, a known period of risk following arthroplasty, nor does it compare full prophylaxis courses extending to 14-35 days.

This retrospective cohort exploited a formulary switch as a natural comparison, with both LMWH groups drawn from the same patient population. Exposure was reliably captured from records, and outcomes were objectively assessed: symptomatic DVT confirmed by duplex ultrasound and bleeding defined clinically and through blood tests. Completeness of follow-up was adequate during the rehabilitation stay, and within-stay outcome data were unlikely to be missed. Strengths included age-adjusted logistic regression for outcomes, offering some confounder control. However, other imbalances (e.g., BMI, length of stay) were not adjusted for, and follow-up ended at hospital discharge, so later VTE or bleeding events were not detected. Overall, the Newcastle-Ottawa scale score was 6/9, suggesting moderate risk of bias, with the main concerns being residual confounding and limited follow-up duration.

Paper 2: Holden and Maceira 2015 [17] was a single-centre retrospective review with the aim of comparing aspirin and anticoagulants used for VTE prophylaxis after THA/TKA. However, it reports agent-level counts within the anticoagulant group, thus allowing an enoxaparin-dalteparin comparison. Adults undergoing THA/TKA at Albany Medical Center between May 1, 2011-July 31, 2013 were identified by ICD-9 codes. VTE events were captured if documented by quality management review or if patients were readmitted to the same institution within 35 days. Patients on therapeutic anticoagulation or with indeterminate prophylaxis were excluded. Anticoagulants included heparin, LMWHs (enoxaparin, dalteparin), fondaparinux, warfarin (INR 1.7-2.3), and rivaroxaban. The average length of stay was 2.7 days, and non-pharmacologic measures were widely used and similar between groups, with no fatal outcomes reported.

Of 1,486 procedures, 85 were excluded, leaving the analytic cohort used for failure-rate calculations. Within the anticoagulant strata, the agent-level table lists: enoxaparin which was given to 153 patients (71 hip, 82 knee), with one (0.65%) patient experiencing a failure in thromboprophylaxis, and dalteparin, which was given to 487 patients (211 hip, 276 knee) and resulted in eight (1.64%) patients suffering from a failure in their VTE prophylaxis.

Failures were calculated as the number of VTEs / the number treated. The study was retrospective and underpowered for modest between-group differences (power analysis suggested ~770 per group for a 2% absolute difference; ~430 per group for 3%). To address baseline-risk imbalance between aspirin and anticoagulants, the authors compared random samples of 100 + 100 for documented VTE risk factors (no difference found). However, this mitigation does not extend to differences among individual anticoagulants.

Absolute symptomatic VTE rates were low for both agents, with a numerically lower rate for enoxaparin compared to dalteparin in this dataset. The paper does not present adjusted comparisons between enoxaparin and dalteparin, and dosing/duration by agent was not reported, limiting causal interpretation. Event capture was restricted to the initial admission and 35-day readmissions to the same hospital, so missed events are plausible.

This single-centre retrospective chart review had several strengths: both exposed and comparison groups were drawn from the same institutional population, exposure (thromboprophylaxis regimen) was ascertained from records, and outcomes (documented postoperative VTE) were captured from readmissions and chart review. Follow-up extended to 35 days, consistent with the typical postoperative VTE risk window.

Weaknesses of this paper include incomplete outcome capture, as VTE events were only recorded if patients were readmitted to the same hospital within 35 days, and VTE events managed elsewhere or beyond 35 days may have been missed. Thus, there is potential under-ascertainment of VTE events. There was also a lack of information provided about dosing regimens and duration for enoxaparin and dalteparin. Regimen variability may influence outcomes, but that would not be possible to assess formally from this study.

Several limitations also reduce the overall Newcastle-Ottawa scale score to 5/9. There was no assurance that patients were VTE-free at baseline, no adjustment for potential confounders beyond demographic similarity checks, and outcome capture depended on return to the same hospital, so VTE treated elsewhere could have been missed. These features introduce the risk of selection and outcome misclassification bias.

Overall, this study is at moderate risk of bias, mainly due to unmeasured confounding and incomplete outcome ascertainment.

Paper 3: Biławicz (2020) [15] was a single-centre, prospective, randomised pilot trial from the Medical University of Warsaw comparing once-daily subcutaneous dalteparin 5000 units and once daily subcutaneous enoxaparin 40 mg for in-hospital VTE prophylaxis in elective THR/TKR. Ethics approval was obtained, but there was no public trial registration. The stated aim was to compare “clinical effectiveness” and biological activity, but outcomes were largely laboratory and peri-operative bleeding proxies, not symptomatic VTE.

The trial included 66 adults (average age 63 ± 12; 44 women, 22 men), randomised to enoxaparin (n = 34) or dalteparin (n = 32). The first prophylactic dose was administered 12 hours pre-op and then once daily; post-op dose ≥12 hours after surgery. All patients received spinal (subarachnoid) anaesthesia with 0.5% Marcaine Spinal Heavy via a 26G needle. Clinical and lab data were collected pre-op, post-op day 1, and day 5.

Outcomes and Measurements

Primary readouts (as reported) were peri-operative bleeding proxies (such as noticed by surgeon/anaesthetist), RBC transfusion (received yes/no and total mL), and coagulation tests including APTT, TT, fibrinogen, Duke’s bleeding time, and Lee-White clotting time. Statistical tests included t/Mann-Whitney for continuous data and χ²/Fisher for categorical; p < 0.05 is significant. No symptomatic VTE outcomes were reported.

Groups were comparable in age/sex/creatinine and most bloods. APTT was slightly longer in dalteparin (32.5 ± 4.0 s vs. 30.4 ± 3.3 s; p = 0.02). Other coagulation tests (thrombin time, fibrinogen, Duke’s, Lee-White) did not differ much at baseline. Operating room assessments, such as surgeon/anaesthetist seeing categorical bleeding, surgical environment, and haemostasis ratings, were similar between groups.

Seventeen (50%) of the patients on enoxaparin required red cell transfusion compared to eight (25%) of the patients on dalteparin (p < 0.05). By our calculation, the relative risk would therefore be 2.00 (95% CI 1.01-3.98), and the risk difference would be +25% (95% CI +2.5% to +47.5%). The total red blood cell volume in mL was also reported with a median of 150 (range 0-600) in the enoxaparin cohort compared to 0 (range 0-1,040) in the dalteparin cohort (p = 0.04).

As for laboratory/coagulation findings, Duke’s bleeding time shortened more on dalteparin and was significantly shorter on day 5: (130 ± 36 s vs. 150 ± 38 s inthe enoxaparin group with p = 0.03). Lee-White coagulation time decreased mildly post-op in both groups. Thrombin time and fibrinogen showed no between-group differences over time in the presented data.

No VTEs were reported in the manuscript (neither symptomatic DVT nor PE within or beyond the five-day window). The authors reference a large observational cohort (~113 k) showing similar VTE and major bleeding rates for enoxaparin versus dalteparin [18], but that evidence is external to this RCT.

With broadly similar lab effects, the higher RBC transfusion use and longer Duke’s bleeding time in the enoxaparin arm suggest a greater risk of blood loss with enoxaparin. The authors connect this to pharmacokinetic differences between LMWHs and call for confirmation in larger studies.

The sample size of 66 was small, and the study is likely to be underpowered when it comes to detecting the respective VTE rates. Follow-up was limited to inpatient stay, and thus, there were no post-discharge events captured and follow-up seems insufficient.

A randomised design was implemented, but sequence generation and allocation concealment were not described, so there is some concern about the RoB here. No blinding of clinicians or outcome assessors was reported. Some key outcomes, such as RBC transfusion decisions and categorical bleeding assessments, were clinician-driven and susceptible to expectation/management bias post-randomisation. As such, there is also a source of bias likely here, but this may be mitigated (albeit with some concerns) by the use of objective haematology (APTT/TT/fibrinogen). The short, in-hospital follow-up and small sample size make attrition unlikely, but withdrawals are not reported, nonetheless. The trial was not registered and did not report symptomatic VTE outcomes despite an aim framed as “clinical effectiveness.” With an emphasis on surrogate/coagulation parameters and peri-operative transfusion without VTE endpoints, there may be a high risk of selective-reporting bias.

Other possible sources of bias include the fact that transfusion thresholds and peri-operative haemostatic management did not seem to be standardised in detail.

Discussion

This review identified three studies directly comparing enoxaparin with dalteparin for VTE prophylaxis in hip and knee arthroplasty: one small RCT - Biławicz et al. (2020) [15] - and two retrospective cohorts - Krotenberg et al. (2001) [16] and Holden and Maceira (2015) [17]. The total sample size was 1,167. Across studies, symptomatic VTE rates were low with both agents and no consistent direction of effect emerged. The randomised trial reported zero symptomatic events in either arm; the rehabilitation cohort observed fewer distal DVTs with dalteparin; the single-centre retrospective review reported fewer symptomatic VTEs with enoxaparin. Overall, the limited and heterogeneous evidence does not indicate a clear difference between enoxaparin and dalteparin, and any potential difference remains uncertain.

The most robust design in the set (the randomised pilot) was underpowered for clinical endpoints and provided only indirect efficacy information (no symptomatic VTE events). The two observational studies point in opposite directions for symptomatic VTE. This inconsistency is more likely due to design and ascertainment differences rather than pharmacological disparity. The rehabilitation cohort only captured rehab-stay, symptom-triggered events (with no post-discharge follow-up), whereas the single-centre review captured events during the index admission and readmissions to the index hospital within 35 days. Neither study performed routine screening, and both were vulnerable to missed events outside their capture windows. Pooling across such heterogeneous designs and follow-up windows would be misleading, so we opted not to meta-analyse VTE outcomes.

Safety data were similarly limited. No major bleeding was reported. The randomised trial observed higher transfusion use with enoxaparin, a finding that aligns with a possible difference in peri-operative haemostasis but remains hypothesis-generating given the small sample size and clinician-driven transfusion decisions. The rehab cohort reported only minor bleeds in small numbers and the single-centre review did not report bleeding outcomes. On balance, available data do not demonstrate a clear difference in major bleeding risk between agents, and transfusion differences should be interpreted cautiously.

The evidence is therefore inconclusive. We identified three main features of the included literature that limit certainty: first is the heterogeneous time windows and settings. One study is rehab-phase only, one is initial-stay and same-centre readmissions and one recorded delivered in-hospital-only prophylaxis but followed patients for three months without routine screening. These differing windows do not align with the known persistence of postoperative VTE risk after discharge, making under-ascertainment likely and comparisons across studies futile. Secondly was lack of exposure detail and standardisation. The RCT specified dosing and timing for both drugs, but the observational studies did not consistently report dose, timing of first dose, or duration of prophylaxis. Without exposure standardisation, confounding by regimen intensity cannot be excluded. And lastly, the risk of bias must also be taken into consideration. The RCT had some concerns (due to unclear allocation concealment, lack of blinding and emphasis on surrogate outcomes). Both observational studies were at serious risk of confounding and outcome ascertainment bias (as there was use of symptom-triggered testing, limited follow-up, and agent choice not being randomised). The single-centre review’s agent-level comparison was secondary to its primary aspirin versus anticoagulants question, further weakening causal inference.

Clinical Implications

Given the limitations above, the most defensible conclusion is that either enoxaparin or dalteparin is reasonable for VTE prophylaxis after hip or knee arthroplasty, with no reliable evidence of superiority for symptomatic VTE prevention or major bleeding. Where institutions already favour one LMWH based on formulary, supply chain, or integration with peri-operative pathways, these data do not compel a change. The historical rehabilitation-phase cost analysis favoured dalteparin; however, current pricing, dispensing fees, and institutional practices may limit generalisability.

Research Implications

A definitive comparison will require an adequately powered, multicentre, randomised, preferably blinded trial that: applies standardised peri-operative protocols, ensures exposure equivalence (clearly defined doses and duration extending into the post-discharge period), uses patient-important primary outcomes (symptomatic DVT/PE and major bleeding), implements more complete follow-up to minimise risk of missed events, registers the protocol, and limits selective reporting. It would also be ideal if a more up-to-date economic evaluation reflecting current costs could be conducted. The lack of such reviews at present may be due to pharmacological similarity, or a shifting prophylactic landscape with the advent of direct oral anticoagulants (DOACs) [19]

Strengths and Limitations of This Review

Strengths include a comprehensive search, explicit eligibility criteria focused on direct head-to-head comparisons, and standardised extraction and risk-of-bias assessment. The primary limitation is the small evidence base (three studies) with methodological heterogeneity that necessitated quantitative synthesis rather than meta-analysis. In addition, outcome definitions and surveillance strategies varied across studies, and two of the three relied on symptomatic-only detection, both of which can bias effect estimates toward no difference.

## Conclusions

Current comparative evidence between enoxaparin and dalteparin in hip and knee arthroplasty is limited and methodologically constrained. Within these constraints, both LMWHs appear to offer similar clinical performance for preventing symptomatic VTE, with no clear, reproducible differences in bleeding. Until higher-quality data are available, agent selection can reasonably be guided by local experience, logistics, and cost, rather than expectations of materially different efficacy or safety.

## Appendices

Database: Ovid MEDLINE(R) and In-Process, In-Data-Review & Other Non-Indexed Citations and Daily <1946 to April 16, 2025> Search Strategy: 1 (Dalteparin Sodium or dalteparin or Fragmin* or Kabi-2165 or Tedelparin or FR-860).ti,ab,kw,kf. (1468) 2 exp Dalteparin/ (946) 3 1 or 2 (1720) 4 (Enox*parin* or Clexane or EMT-966 or EMT-967 or Lovenox or PK-10,169 PK-10169 or Xaparin or arovi or inhixa or axberi or exaranre or enoxapo or iclunox or noromby or redesca or thorinane or anoxaparine or enoxaparinum natricum or enoxaparina sodica or enoxaparine sodique).ti,ab,kw,kf. (5946) 5 exp Enoxaparin/ (4103) 6 4 or 5 (6949) 7 (Venous Thromboembolism prophylaxis or VTE prophylaxis or thromboprophylaxis or thrombosis prevention or venous thrombosis prevention or dvt prophylaxis or deep vein prophylaxis or anti-thrombotic prophylaxis).ti,ab,kw,kf. (10742) 8 exp Venous Thromboembolism/ (17711) 9 7 or 8 (23673) 10 3 and 6 and 9 (123) 11 (knee arthroplast* or knee replacement arthroplast* or knee replacement).ti,ab,kw,kf. (45193) 12 exp Arthroplasty, Replacement, Knee/ (35409) 13 11 or 12 (50911) 14 (hip arthroplast* or hip replacement arthroplast* or hip replacement).ti,ab,kw,kf. (44962) 15 exp Arthroplasty, Replacement, Hip/ (37933) 16 14 or 15 (56375) 17 3 and 6 and 9 and 13 and 16 (18)

Database: Embase <1974 to 2025 April 16> Search Strategy: 1 (Dalteparin Sodium or dalteparin or Fragmin* or Kabi-2165 or Tedelparin or FR-860).ti,ab,kw,kf. (2393) 2 exp dalteparin/ (9047) 3 1 or 2 (9304) 4 (Enox*parin* or Clexane or EMT-966 or EMT-967 or Lovenox or PK-10,169 PK-10169 or Xaparin or arovi or inhixa or axberi or exaranre or enoxapo or iclunox or noromby or redesca or thorinane or anoxaparine or enoxaparinum natricum or enoxaparina sodica or enoxaparine sodique).ti,ab,kw,kf. (12582) 5 exp enoxaparin/ (36709) 6 4 or 5 (37496) 7 (Venous Thromboembolism prophylaxis or VTE prophylaxis or thromboprophylaxis or thrombosis prevention or venous thrombosis prevention or dvt prophylaxis or deep vein prophylaxis or anti-thrombotic prophylaxis).ti,ab,kw,kf. (18608) 8 exp thrombosis prevention/ (19649) 9 exp venous thromboembolism/ (222626) 10 7 or 8 or 9 (234917) 11 3 and 6 and 10 (3016) 12 (knee arthroplast* or knee replacement arthroplast* or total knee arthroplast* or knee replacement).ti,ab,kw,kf. (55014) 13 exp knee arthroplasty/ (65141) 14 12 or 13 (69926) 15 (hip arthroplast* or hip replacement arthroplast* or total hip arthroplast* or hip replacement).ti,ab,kw,kf. (54299) 16 exp hip arthroplasty/ (44133) 17 15 or 16 (69853) 18 3 and 6 and 10 and 14 and 17 (127)
